# Supplementary material for: The desensitization pathway of GABAA receptors, one subunit at a time
Source: Nat Commun. 2020 Oct 23;11:5369. doi: 10.1038/s41467-020-19218-6 (PMC7585415; doi:10.1038/s41467-020-19218-6)
Supplement: Supplementary file 1 — Supplementary Information [file 41467_2020_19218_MOESM1_ESM.pdf]

The desensitization pathway of $GABA_A$ receptors, one subunit at a time

Marc Gielen, Nathalie Barilone & Pierre-Jean Corringer

Supplementary Information

SUPPLEMENTARY DISCUSSION

Model III: limitations and implications for single-channel recordings

We have to acknowledge here two main shortfalls of our model. First, no unliganded desensitized state is present, although these are known to exist and contribute to the recovery from desensitization upon removal of the agonist[1]. Our kinetic model thus cannot be used to model recovery from desensitization. Second, at the single channel level, our model makes a strong prediction when the channel transits from the open state to a more stable pre-desensitized state still capable of conducting ions. Indeed, in the open state, single-channel openings on the ms timescale are separated by brief shut times reflecting sojourns in resting or pre-active states[2], giving rise to a maximum open probability of about 70%-80% for α1β2γ2 $GABA_A$Rs[3]. However, once the channel enters a pre-desensitized state in our final scheme – i.e., AD_3, AD_4 or AD_5 -, the predicted lifetime of such state should produce uninterrupted single-channel openings of several seconds for wild-type receptors. Such openings are not observed in single-channel recordings. It is likely that, when one subunit enters its desensitized conformation, the other subunits retain the ability to visit their resting conformation, thereby providing a way to maintain an open probability well below unity. Still, one could expect a strong influence of the receptor's desensitization on its single-channel gating efficacy under equilibrium conditions, e.g. in cell-attached patch-clamp recordings. It is thus striking that a recent study highlighted such phenomenon at α1 glycine receptors (GlyRs)[4]. Indeed, Ivica *et al.* showed that deletion of the intracellular domain (ICD) of homomeric human α1 GlyRs largely increases their efficacy of gating. At the same time, the ICD is known to affect the desensitization of α1 GlyRs[5–7], and the study by Ivica *et al.* displays single-channel clusters that seem largely lengthened by the ICD deletion, suggesting that their ICD-deleted construct shows reduced levels of desensitization (comparing the

glycine and alanine recordings from panels from Figure 2B and Figure 3B in ref[4]). It is thus tempting to speculate that the ICD-deleted GlyRs constructs from the study by Ivica *et al.* show a greater stability of their pre-desensitized states with one single subunit being desensitized, resulting in both lengthened single-channel clusters and higher cluster open probability under equilibrium conditions.

Our model makes another prediction regarding single-channel recordings, which would provide strikingly different estimation of desensitization kinetics under equilibrium versus non-equilibrium conditions. Indeed, with cell-attached recordings performed in the continued presence of the agonist, some desensitized channels would spontaneously recover and visit a conducting state, amenable to electrophysiological detection. Given our set of parameters, the kinetically favoured pathway would be for a receptor in a AD_{345} state to transit to the AD_{34} state and then to the conducting pre-desensitized AD_3 state. From there, the favoured transition would be to re-enter the AD_{34} state with a microscopic rate of $\gamma.\delta^+$, i.e. 28 s^{-1}, resulting in a cluster duration of about 36 ms. On the contrary, a receptor recorded in non-equilibrium conditions, such as performed in fast-perfusion experiments in the outside-out configuration or in whole-cell experiments, would start off in the resting state R, transit to the agonist-bound AR state, and then finally open (AO state). The electrophysiological current would be detectable for the entire duration from the entry to the open state to the exit in a fully desensitized state. In this case, the favoured routes would either be AO to AD_4 to AD_{45} or AO to AD_5 to AD_{45}. The apparent corresponding macroscopic rate would roughly equate to $2.(\delta^+/\delta^-).\delta^+$, i.e. about 0.25 s^{-1}, resulting in a cluster duration of about 4 s. Thus, our model suggests that cell-attached patch-clamp recordings might not be predictive of the desensitization properties of $\alpha1\beta2\gamma2$ $GABA_ARs$ driven by a sudden rise in agonist concentration, as observed during phasic synaptic release. Of note, a recent study on NMDA receptors has shown the existence of two distinct preactivated / primed states by analysing the

opening latency ("time to open") in single-channel recordings, which were obtained with fast-perfusion of outside-out patches containing only one channel[8]. This study highlights the importance to study ligand-gated ion channels under non-equilibrium conditions, in order to gain insights in their gating pathways.

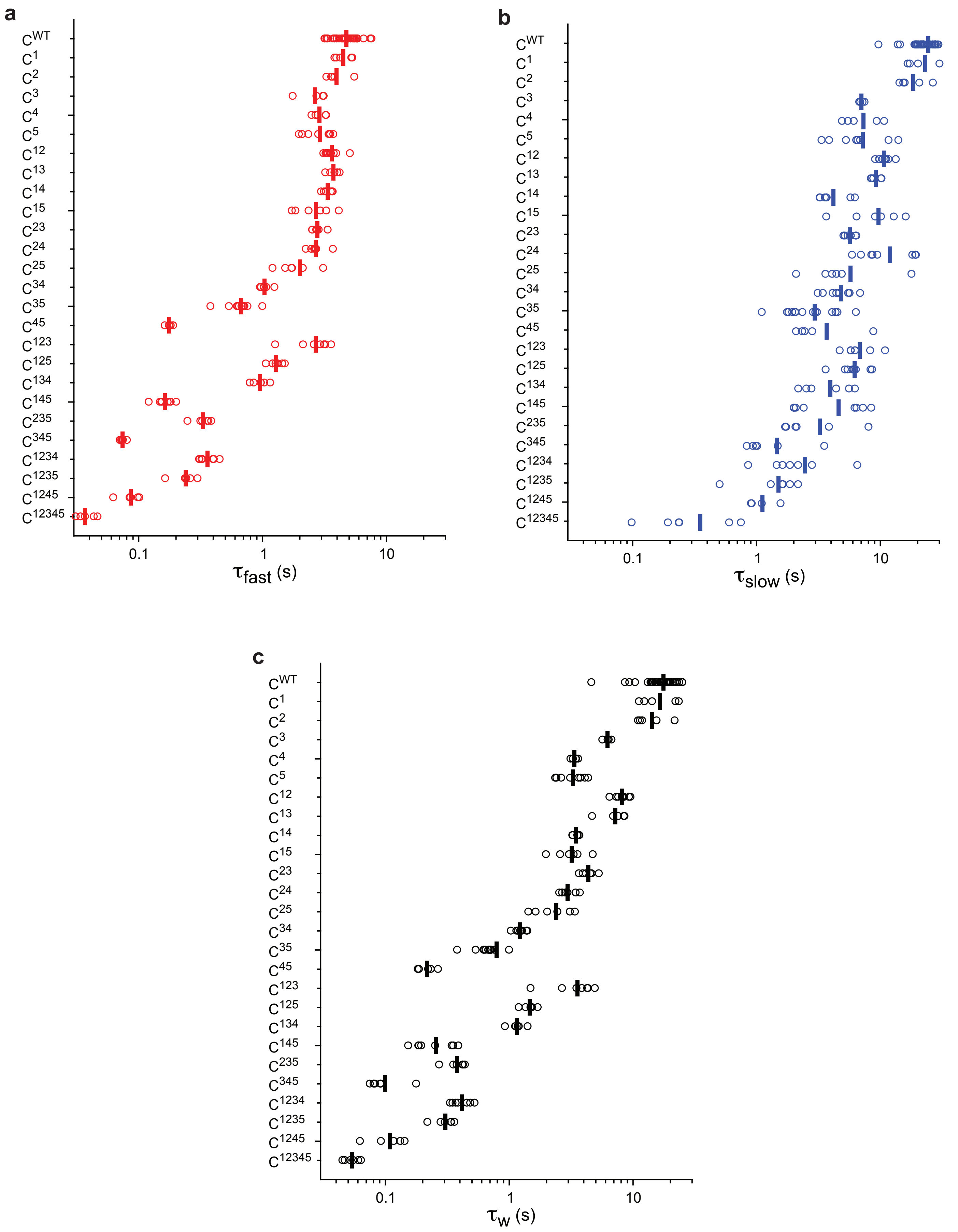

Supplementary Figure 1. Individual data points for desensitization decay time constants. a-c, Individual data points and means are shown as circles bars, respectively. **a,** Data for fast desensitization time constants (red). **b,** Data for slow desensitization time constants (blue). **c,** Data for weighted desensitization time constants (black). See Supplementary Table 1 for the numerical mean values, the number of cells and the number of independent experiments.

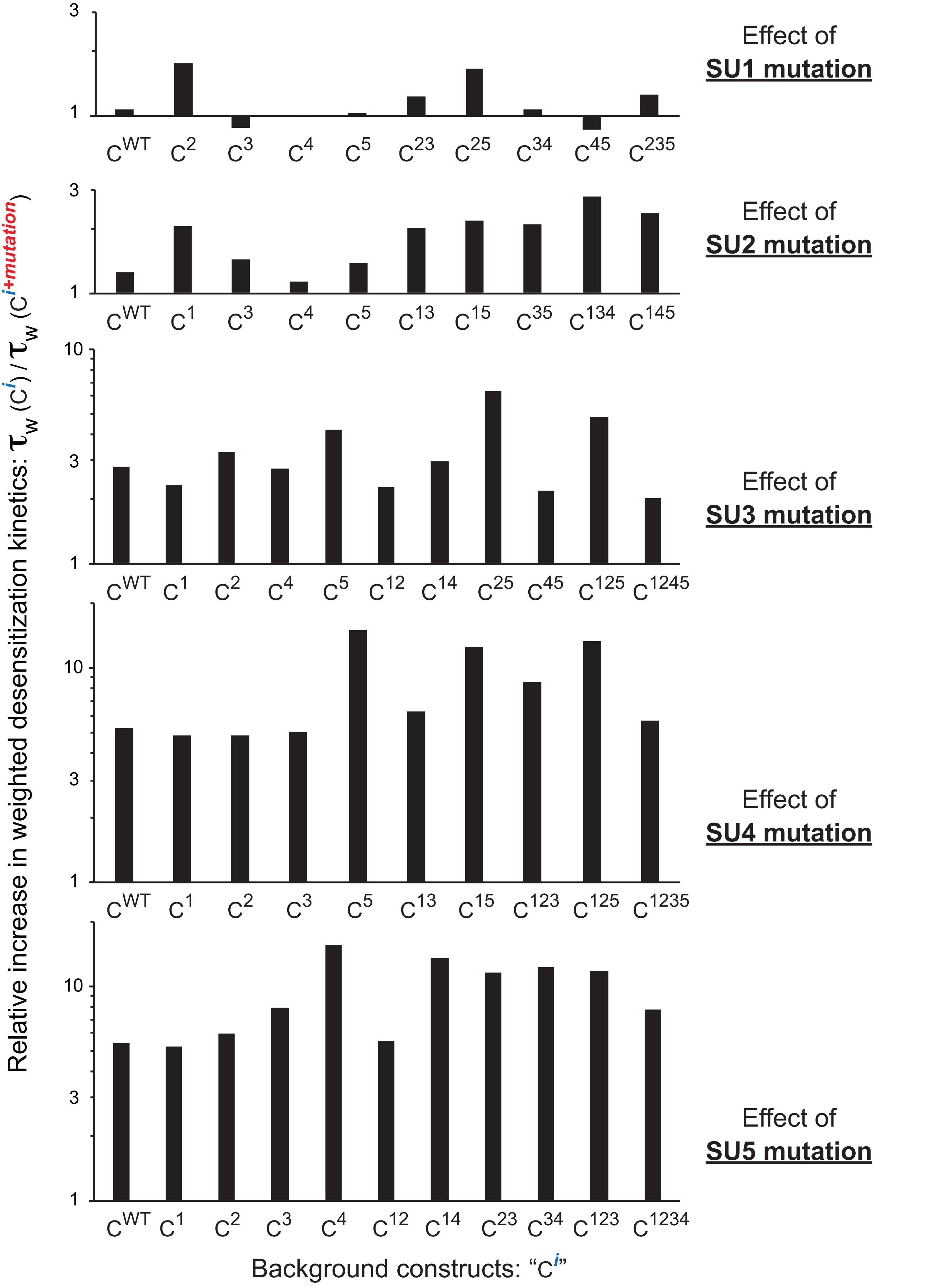

Supplementary Figure 2. Relative effect of M3-5' mutations on the weighted desensitization kinetics of a series of background mutated concatemers. Each bar plot corresponds to the relative effect of the M3-5' mutation of the indicated subunit on the weighted desensitization rate of the background constructs. Values were calculated as the ratio between corresponding mean τ_W values.

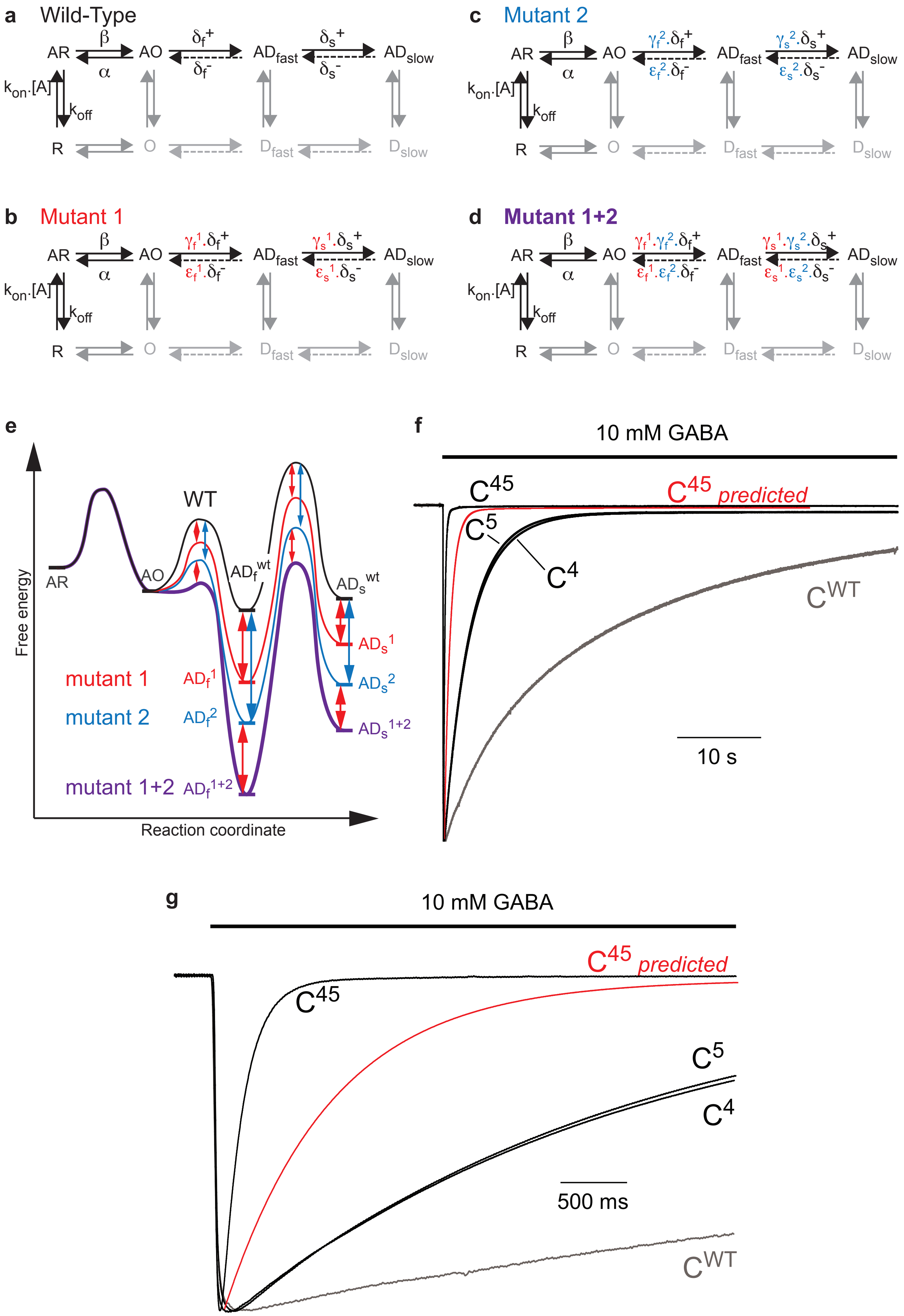

a Wild-Type
b Mutant 1
c Mutant 2
d Mutant 1+2
AR
AO
ADfast
ADslow
R
O
Dfast
Dslow
β
α
kon.[A]
koff
e
WT
Free energy
Reaction coordinate
mutant 1
mutant 2
mutant 1+2
f
10 mM GABA
C45
C45 predicted
C5
C4
CWT
10 s
g
10 mM GABA
500 ms

Supplementary Figure 3. A concerted model only including pseudo-symmetrical states cannot account for the synergistic effects of SU4 and SU5 M3-5' valine mutations. a-d, Simplified concerted kinetic schemes used to model wild-type (panel **a**), single mutant (panels **b** & **c**) and double mutant (panel **d**) concatemers. While unliganded openings are possible at all ligand-gated receptors, consistent with a Monod-Wyman-Changeux model of allosteric proteins, they are exceedingly rare at wild-type α1β2γ2 $GABA_A$Rs (see Main Text), and we therefore decided not to include them in our kinetic modelling. In the absence of direct interaction between the mutated residues, mutations are expected to have additive effects (see Main Text) as described in panel **d**. **e,** Putative free energy diagram translating the kinetic schemes from panels **a-d**. **f-g,** Representative currents (in black) from C^{WT}, C^4, C^5 and C^{45} constructs are overlaid with the simulated C^{45} response (in red) as predicted from the concerted model adjusted to account for the wild-type and single mutant constructs (C^{WT}, C^4 and C^5). Note that the concerted model largely underestimates the desensitization kinetics of the double mutant C^{45}.

a

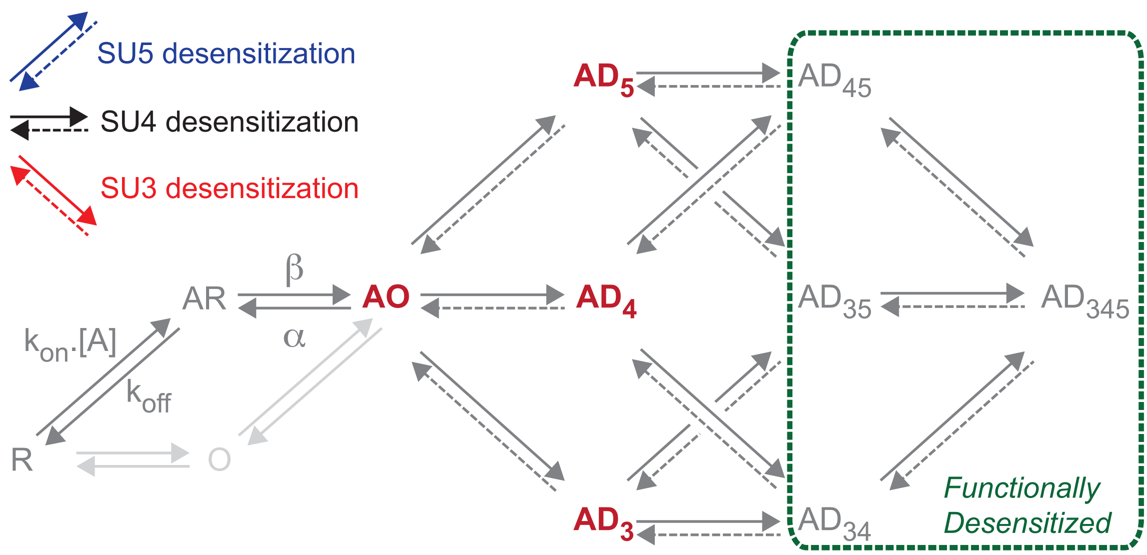

b

		wild-type	*SU3 mutant*	*SU4 mutant*	*SU5 mutant*
**SU3**	*desensitization rate*	δ_3^+	$c_3^+.\delta_3^+$	δ_3^+	δ_3^+
	recovery rate	δ_3^-	δ_3^-	δ_3^-	δ_3^-
**SU4**	*desensitization rate*	δ^+	δ^+	$c_4^+.\delta^+$	δ^+
	recovery rate	δ^-	δ^-	δ^-	δ^-
**SU5**	*desensitization rate*	δ^+	δ^+	δ^+	$c_5^+.\delta^+$
	recovery rate	δ^-	δ^-	δ^-	δ^-

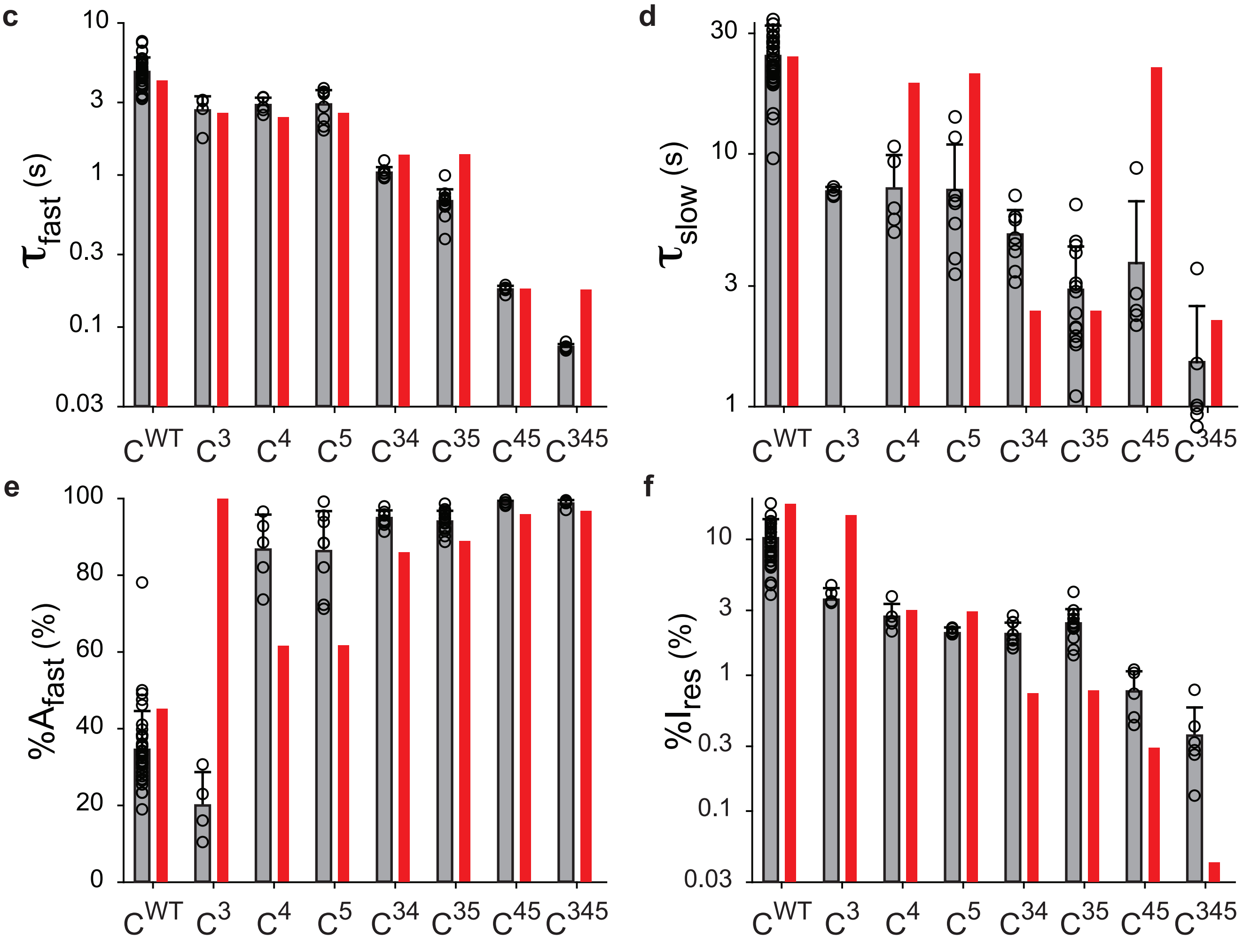

Supplementary Figure 4. Model II': underestimated values for the residual current in the absence of mutant-induced effects on desensitization recovery. a, For wild-type receptors, Model II and Model II' are strictly identical (see Main Figure 5). **b,** For mutated concatemers, Model II' incorporates the hypothesis that the mutations do not affect desensitization recovery rates. **c-f,** Bar graphs summarizing the experimental data (in black) vs the simulations (in red) for the indicated concatemers on the kinetics of the fast (panel **a**) and slow (panel **b**) desensitization components, the relative amplitude of the fast component (panel c) and the residual current after a 1 min long application of 10 mM GABA (panel **d**). Note that all simulated multiple mutants display largely reduced residual current as compared with experimental data. Experimental data are shown as means (bar graphs) and standard deviations (error bars), with individual data points indicated as circles. See Supplementary Table 1 for numerical values, number of cells and number of independent experiments.

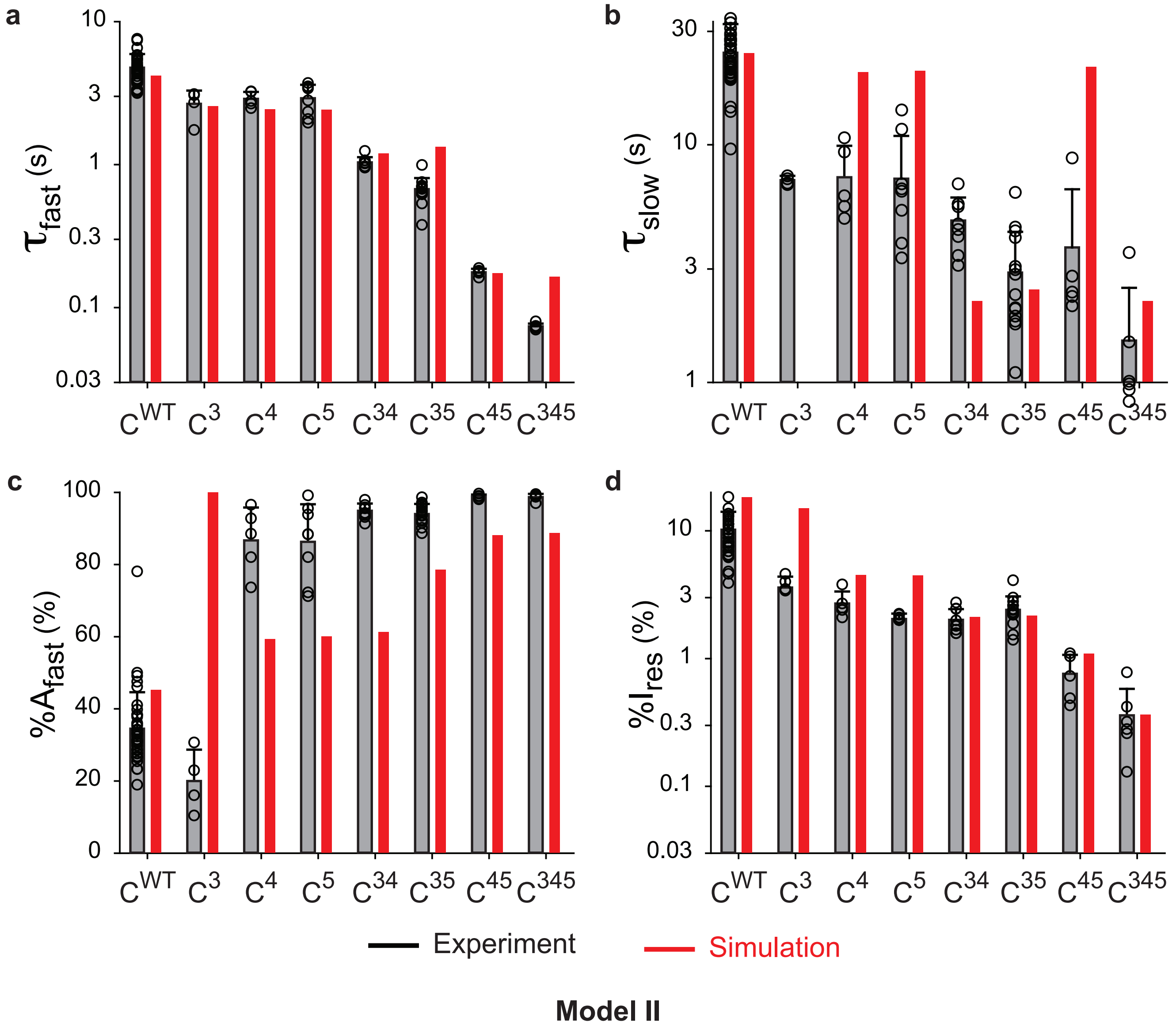

Supplementary Figure 5. Simulations from Model II. a-d, Bar graphs summarizing the experimental data (in black) vs the simulations (in red) for the indicated concatemers on the kinetics of the fast (panel **a**) and slow (panel **b**) desensitization components, the relative amplitude of the fast component (panel **c**) and the residual current after a 1 min long application of 10 mM GABA (panel **d**). See Supplementary Table 3 for the numerical values of parameters. Experimental data are shown as means (bar graphs) and standard deviations (error bars), with individual data points indicated as circles. See Supplementary Table 1 for numerical values, number of cells and number of independent experiments.

a

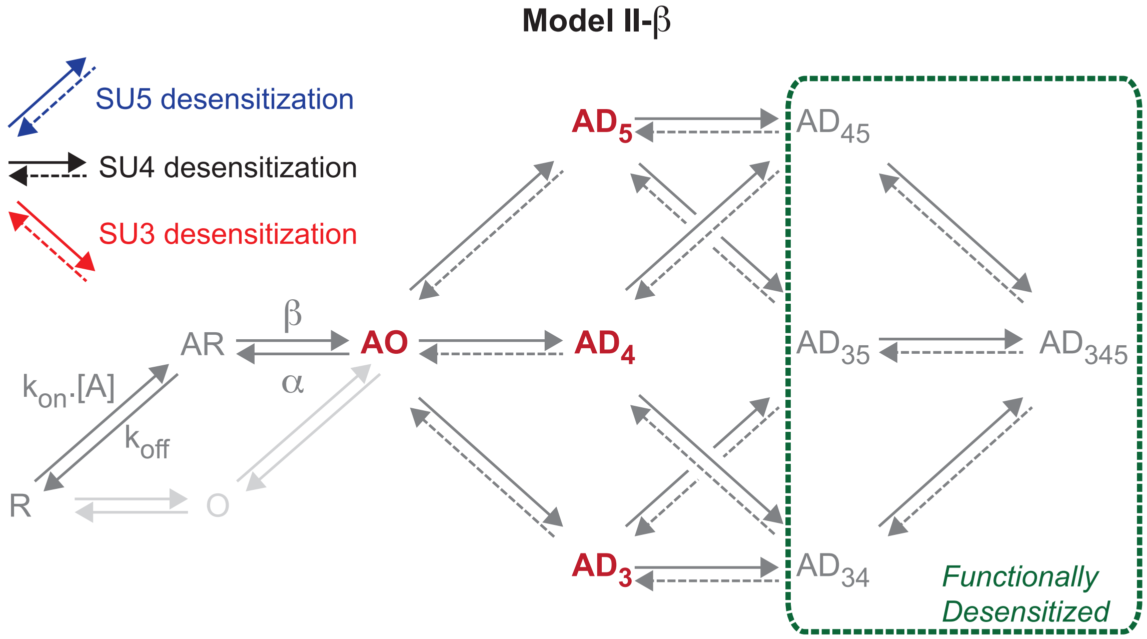

b

		wild-type	*SU3 mutant*	*SU4 mutant*	*SU5 mutant*
**SU3**	*desensitization rate*	δ_3^+	$c_3^+.\delta_3^+$	δ_3^+	δ_3^+
	recovery rate	δ_3^-	$c_3^-.\delta_3^-$	δ_3^-	δ_3^-
**SU4**	*desensitization rate*	δ^+	$c_{34}^+.\delta^+$	$c_4^+.\delta^+$	δ^+
	recovery rate	δ^-	$c_{34}^-.\delta^-$	$c_4^-.\delta^-$	δ^-
**SU5**	*desensitization rate*	δ^+	δ^+	δ^+	$c_5^+.\delta^+$
	recovery rate	δ^-	δ^-	δ^-	$c_5^-.\delta^-$

Supplementary Figure 6. Model II-β: mutation of SU3 directly impacts on SU4 desensitization. a, For wild-type receptors, Model II and Model II-β are strictly identical. **b,** For mutated concatemers, Model II-β incorporates the hypothesis that the mutation of SU3 also affects the desensitization of SU4 by increasing both its desensitization and recovery rates, by ratios c_{34}^+ and c_{34}^-, respectively.

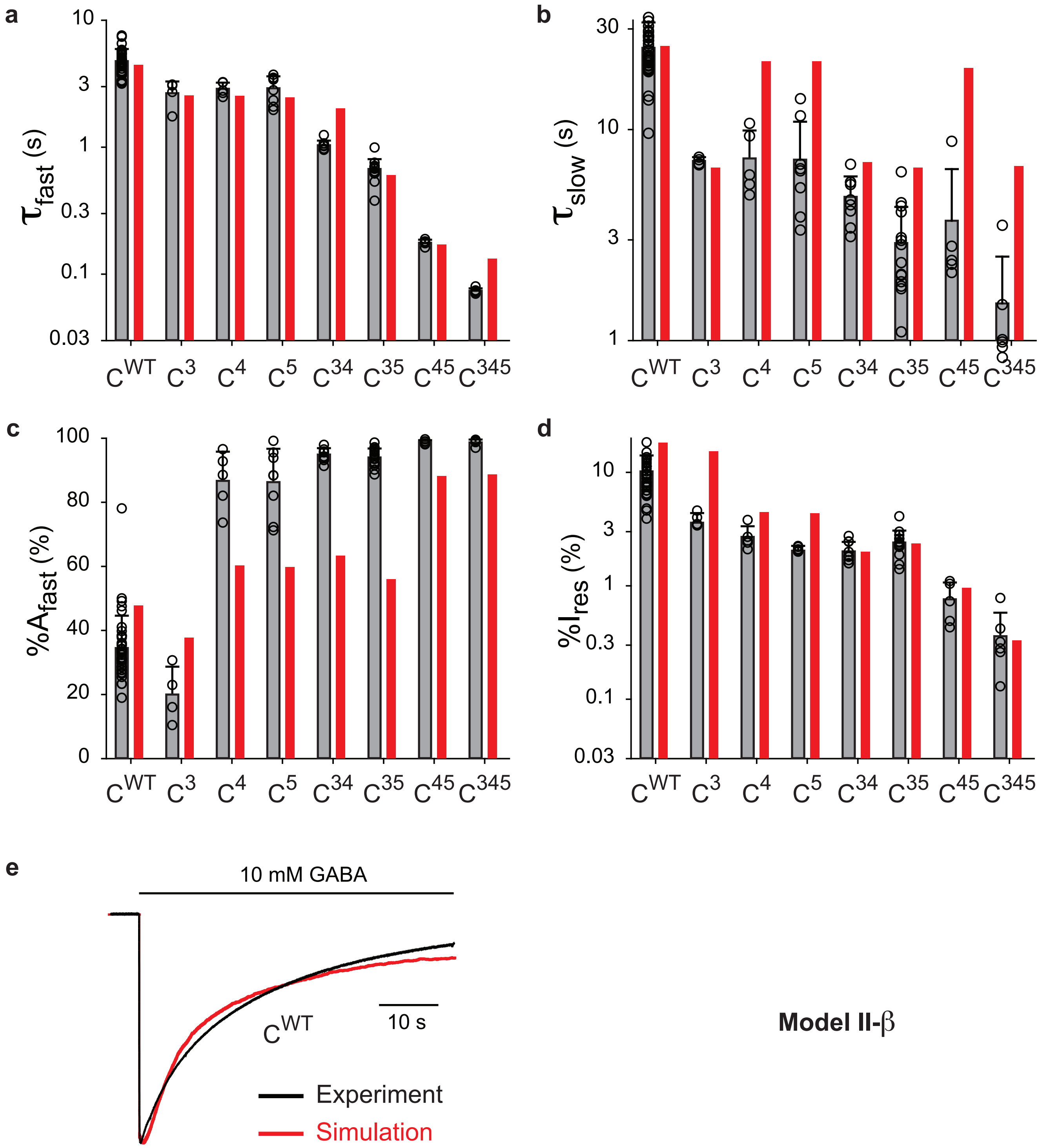

Supplementary Figure 7. Simulations from Model II-β. a-d, Bar graphs summarizing the experimental data (in black) vs the simulations (in red) for the indicated concatemers on the kinetics of the fast (panel **a**) and slow (panel **b**) desensitization components, the relative amplitude of the fast component (panel **c**) and the residual current after a 1 min long application of 10 mM GABA (panel **d**). See Supplementary Table 3 for the numerical values of parameters. Experimental data are shown as means (bar graphs) and standard deviations (error bars), with individual data points indicated as circles. See Supplementary Table 1 for numerical values, number of cells and number of independent experiments. **e,** Overlay of a representative current for C^{WT} (in black) with the response simulated according to Model II-β (in red). Note that both the amplitude of the fast desensitization component and residual currents are overestimated in the simulation.

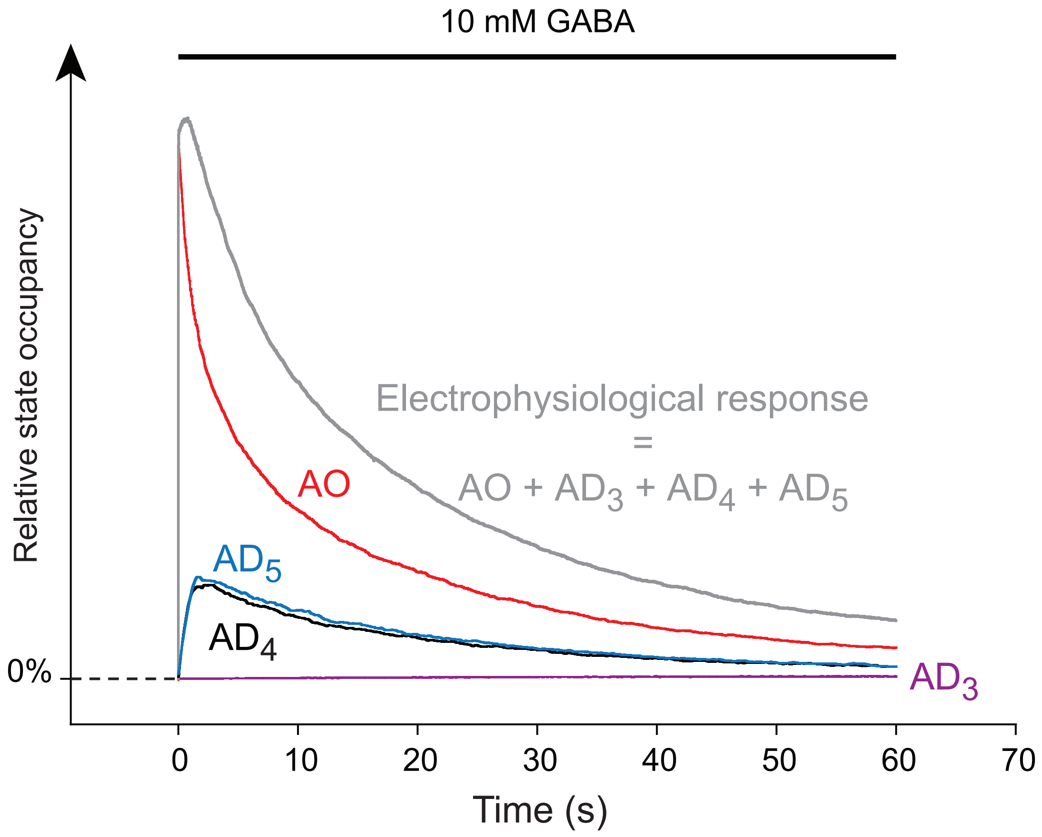

Supplementary Figure 8. Time-dependant changes in the occupancy of the receptor's states as predicted by model III. The relative occupancies of each state contributing to the electrophysiological currents, namely AO, AD_3, AD_4 and AD_5, are compared to the overall population of receptors in an active conformation (AO + AD_3 + AD_4 + AD_5), which directly translates the experimental recordings. The analysis of states occupancies was performed with QuB simulations.

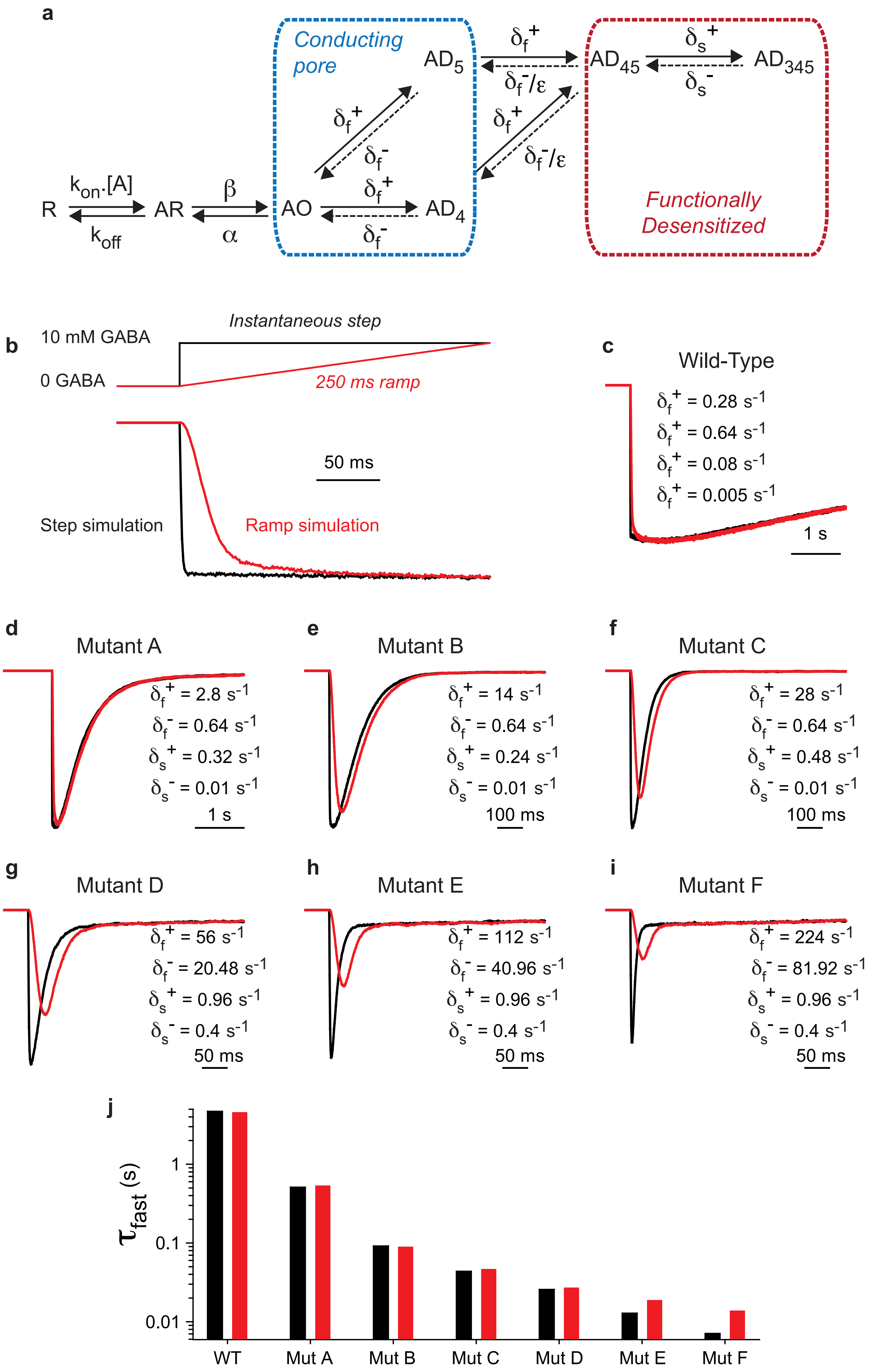
a
Conducting pore
Functionally Desensitized
R
AR
AO
AD4
AD5
AD45
AD345
kon.[A]
koff
β
α
δf+
δf-
δf-/ε
δs+
δs-
b
10 mM GABA
0 GABA
Instantaneous step
250 ms ramp
50 ms
Step simulation
Ramp simulation
c
Wild-Type
δf+ = 0.28 s-1
δf+ = 0.64 s-1
δf+ = 0.08 s-1
δf+ = 0.005 s-1
1 s
d
Mutant A
δf+ = 2.8 s-1
δf- = 0.64 s-1
δs+ = 0.32 s-1
δs- = 0.01 s-1
1 s
e
Mutant B
δf+ = 14 s-1
δf- = 0.64 s-1
δs+ = 0.24 s-1
δs- = 0.01 s-1
100 ms
f
Mutant C
δf+ = 28 s-1
δf- = 0.64 s-1
δs+ = 0.48 s-1
δs- = 0.01 s-1
100 ms
g
Mutant D
δf+ = 56 s-1
δf- = 20.48 s-1
δs+ = 0.96 s-1
δs- = 0.4 s-1
50 ms
h
Mutant E
δf+ = 112 s-1
δf- = 40.96 s-1
δs+ = 0.96 s-1
δs- = 0.4 s-1
50 ms
i
Mutant F
δf+ = 224 s-1
δf- = 81.92 s-1
δs+ = 0.96 s-1
δs- = 0.4 s-1
50 ms
j
τfast (s)
1
0.1
0.01
WT
Mut A
Mut B
Mut C
Mut D
Mut E
Mut F

Supplementary Figure 9. TEVC currents rise-time allow the measurement of fast desensitization kinetics down to the 25 ms timescale. a, Simplified kinetic scheme used to simulate receptors' desensitization, according to the kinetically favoured pathway from Main Figure 7. See Supplementary Table 3 for k_{on}, k_{off}, α and β numerical values. **b,** Simulating a gradual solution exchange with a 250 ms ramp protocol (red trace) yields a 20-80% current rise time of 23 ms, akin the experimentally achieved values, to be compared with a simulated instantaneous step solution exchange (black trace). **c-i,** Comparison between currents simulated with step (black traces) and ramp (red traces) application protocols, for a series of wild-type and virtual mutant receptors simulated with the indicated desensitization and recovery rates. Note the difference in peak current amplitude for Mutants C-F. **j,** Bar graph showing the time constants for the fast desensitization component of the virtual constructs simulated with step (black bars) and ramp (red bars). Note the discrepancy between the values from step and ramp protocols when desensitization occurs on a timescale faster than 20 ms: Mutant D displays τ_{fast} values of 25.7 ms and 27.1 ms with step and ramp protocols, respectively, while Mutant E shows τ_{fast} values of 12.8 ms and 18.7 ms with step and ramp protocols, respectively. The extreme case in the series, Mutant F, shows τ_{fast} values of 7.1 ms and 13.8 ms with step and ramp protocols, respectively.

Construct	SU1	SU2	SU3	SU4	SU5	I_{peak} (µA)		τ_{fast} (s)	τ_{slow} (s)	%A_{fast}	τ_w (s)	%I_{res}	# cells	# animals
C^{WT}	WT	WT	WT	WT	WT	1.71	[1.05;2.8]	4.8 ± 1.2	24.4 ± 7.8	34.5% ± 10.1%	17.6 ± 5.1	10.2% ± 3.9%	n = 40	N = 17
C^{1}	mut	WT	WT	WT	WT	3.24	[2.00;5.73]	4.5 ± 0.7	23.0 ± 7.0	35.9% ± 4.9%	16.6 ± 5.7	9.1% ± 3.4%	n = 5	N = 2
C^{2}	WT	mut	WT	WT	WT	3.35	[0.46;164]	4.0 ± 0.9	18.5 ± 5.1	29.9% ± 5.5%	14.2 ± 4.5	7.5% ± 2.9%	n = 5	N = 2
C^{3}	WT	WT	mut	WT	WT	1.42	[0.27;26]	2.7 ± 0.6	7.1 ± 0.3	20.0% ± 8.7%	6.2 ± 0.4	3.6% ± 0.8%	n = 4	N = 2
C^{4}	WT	WT	WT	mut	WT	1.18	[0.28;11]	2.9 ± 0.3	7.3 ± 2.6	86.7% ± 9.1%	3.4 ± 0.2	2.7% ± 0.7%	n = 5	N = 2
C^{5}	WT	WT	WT	WT	mut	0.86	[0.34;3.21]	2.9 ± 0.7	7.2 ± 3.7	86.3% ± 10.4%	3.3 ± 0.8	2.0% ± 0.2%	n = 8	N = 4
C^{12}	mut	mut	WT	WT	WT	0.91	[0.44;1.87]	3.6 ± 0.6	10.7 ± 1.2	35.8% ± 8.1%	8.1 ± 0.9	2.8% ± 0.2%	n = 10	N = 4
C^{13}	mut	WT	mut	WT	WT	1.21	[0.32;6.61]	3.7 ± 0.4	9.2 ± 0.9	36.9% ± 19.7%	7.2 ± 1.6	3.5% ± 0.5%	n = 5	N = 2
C^{14}	mut	WT	WT	mut	WT	1.68	[0.73;6.09]	3.3 ± 0.3	4.2 ± 1.2	60.8% ± 18.6%	3.4 ± 0.2	2.7% ± 1.0%	n = 7	N = 2
C^{15}	mut	WT	WT	WT	mut	2.05	[0.48;17.2]	2.7 ± 0.9	9.7 ± 4.4	83.4% ± 32.1%	3.2 ± 0.9	2.5% ± 0.6%	n = 6	N = 3
C^{23}	WT	mut	mut	WT	WT	1.75	[0.53;18.9]	2.8 ± 0.3	5.7 ± 0.6	45.6% ± 18.2%	4.3 ± 0.6	3.3% ± 0.3%	n = 6	N = 2
C^{24}	WT	mut	WT	mut	WT	3.09	[1.15;12.7]	2.7 ± 0.4	12.0 ± 5.8	95.7% ± 5.5%	3.0 ± 0.4	2.2% ± 0.6%	n = 8	N = 2
C^{25}	WT	mut	WT	WT	mut	0.58	[0.33;1.13]	2.0 ± 0.7	5.7 ± 5.1	80.8% ± 25.0%	2.4 ± 0.7	1.9% ± 0.8%	n = 7	N = 3
C^{34}	WT	WT	mut	mut	WT	0.60	[0.3;1.77]	1.0 ± 0.1	4.8 ± 1.2	94.9% ± 2.0%	1.2 ± 0.1	2.0% ± 0.4%	n = 9	N = 3
C^{35}	WT	WT	mut	WT	mut	1.31	[0.68;2.81]	0.67 ± 0.13	2.9 ± 1.4	94.0% ± 2.8%	0.79 ± 0.15	2.4% ± 0.7%	n = 14	N = 6
C^{45}	WT	WT	WT	mut	mut	2.64	[0.59;40.8]	0.18 ± 0.01	3.7 ± 2.8	98.8% ± 0.6%	0.22 ± 0.03	0.8% ± 0.3%	n = 5	N = 2
C^{123}	mut	mut	mut	WT	WT	3.48	[1.62;9.94]	2.7 ± 0.8	6.8 ± 2.1	71.0% ± 20.1%	3.6 ± 1.2	3.6% ± 1.1%	n = 7	N = 2
C^{125}	mut	mut	WT	WT	mut	0.30	[0.18;0.55]	1.3 ± 0.1	6.2 ± 1.6	96.1% ± 1.8%	1.5 ± 0.1	1.7% ± 0.4%	n = 8	N = 2
C^{134}	mut	WT	mut	mut	WT	0.32	[0.14;0.69]	0.96 ± 0.13	3.9 ± 1.7	92.1% ± 3.5%	1.2 ± 0.2	2.5% ± 0.7%	n = 7	N = 2
C^{145}	mut	WT	WT	mut	mut	0.43	[0.22;0.9]	0.16 ± 0.02	4.6 ± 2.7	98.0% ± 0.7%	0.26 ± 0.09	0.6% ± 0.3%	n = 8	N = 2
C^{235}	WT	mut	mut	WT	mut	1.26	[0.87;1.86]	0.33 ± 0.05	3.2 ± 2.5	97.8% ± 1.2%	0.38 ± 0.06	1.1% ± 0.4%	n = 6	N = 3
C^{345}	WT	WT	mut	mut	mut	1.61	[0.68;7.13]	0.07 ± 0.003	1.5 ± 1.0	98.7% ± 0.9%	0.10 ± 0.04	0.4% ± 0.2%	n = 6	N = 2
C^{1234}	mut	mut	mut	mut	WT	0.86	[0.15;10.1]	0.36 ± 0.057	2.5 ± 1.9	96.0% ± 3.6%	0.41 ± 0.07	1.4% ± 0.4%	n = 7	N = 2
C^{1235}	mut	mut	mut	WT	mut	1.19	[0.17;34.6]	0.24 ± 0.044	1.5 ± 0.6	93.4% ± 4.9%	0.31 ± 0.05	1.3% ± 1.2%	n = 6	N = 2
C^{1245}	mut	mut	WT	mut	mut	0.35	[0.19;0.67]	0.09 ± 0.015	1.1 ± 0.3	97.4% ± 2.2%	0.11 ± 0.03	0.5% ± 0.2%	n = 5	N = 2
C^{12345}	mut	mut	mut	mut	mut	0.57	[0.28;1.37]	0.04 ± 0.007	0.4 ± 0.3	89.5% ± 10.7%	0.05 ± 0.01	0.5% ± 0.3%	n = 6	N = 2

SU1 mut & SU3 mut = β_2^{N303V} ; SU2 mut & SU4 mut = α_1^{N307V} ; SU5 mut = γ_2^{H318V}

Supplementary Table 1. Kinetics and extent of desensitization of the pentameric $GABA_A$ concatemers used in the present study. Except for peak currents, all data are shown as means ± standard deviations. For each construct, n is the number of cells (*Xenopus laevis* oocytes) that we recorded, and N is the number of animals (i.e. number of independent *Xenopus* ovaries) that we used for all constructs - i.e. n cells recorded over N series of independent experiments. For peak currents, arithmetic means and 95% confidence interval are displayed, assuming log-normal distribution of currents and using the Cox method for confidence intervals[9].

Parameter	Value	Unit
k_{on}	1.00E+05	$M^{-1}.s^{-1}$
k_{off}	100	s^{-1}
β	2000	s^{-1}
α	500	s^{-1}
δ_f^+	0.12	s^{-1}
δ_f^-	0.08	s^{-1}
δ_s^+	0.1	s^{-1}
δ_s^-	0.01	s^{-1}
γ_f^4	3.5	
ε_f^4	0.5	
γ_s^4	1	
ε_s^4	3	
γ_f^5	3.5	
ε_f^5	0.5	
γ_s^5	1	
ε_s^5	3	

Concerted model parameters

Supplementary Table 2. Numerical values used for the parameters from the concerted model in Supplementary Figure 2.

Parameter	Model Ia	Model Ib	Model II	Model II-β	Model III	Unit
k_{on}	1.0 E+05	1.0 E+05	1.0 E+05	1.0 E+05	1.0 E+05	$M^{-1}.s^{-1}$
k_{off}	100	100	100	100	100	s^{-1}
β	2000	2000	2000	2000	2000	s^{-1}
α	500	500	500	500	500	s^{-1}
δ^+	0.035	0.035	0.24	0.24	0.28	s^{-1}
δ^-	0.106	0.106	0.13	0.13	0.64	s^{-1}
δ_3^+	0.042	0.042	0.05	0.05	8.0 E-04	s^{-1}
δ_3^-	1.05 E-03	1.05 E-03	3.0 E-04	3.0 E-04	5.0 E-03	s^{-1}
γ	-	-	-	-	100	
ε	-	-	-	-	10	
c_3^+	4	10	10	3	3	
c_3^-	-	-	1	2	2	
c_{34}^+	-	-	-	4	4	
c_{34}^-	-	-	-	4	3	
c_4^+	10	100	30	30	20	
c_4^-	-	-	3	3	1	
c_5^+	10	100	30	30	60	
c_5^-	-	-	3	3	1	

Supplementary Table 3. Numerical values used for the parameters from the non concerted models I, II, II-β and III.

1. Gielen, M. & Corringer, P.-J. The dual-gate model for pentameric ligand-gated ion channels activation and desensitization. *J. Physiol.* **596**, 1873–1902 (2018).

2. Lape, R., Colquhoun, D. & Sivilotti, L. G. On the nature of partial agonism in the nicotinic receptor superfamily. *Nature* (2008) doi:10.1038/nature07139.

3. Mortensen, M. *et al.* Activation of single heteromeric GABA(A) receptor ion channels by full and partial agonists. *J. Physiol.* **557**, 389–413 (2004).

4. Ivica, J. *et al.* The intracellular domain of homomeric glycine receptors modulates agonist efficacy. *J. Biol. Chem.* jbc.RA119.012358 (2020) doi:10.1074/jbc.RA119.012358.

5. Breitinger, H.-G. *et al.* Molecular dynamics simulation links conformation of a pore-flanking region to hyperekplexia-related dysfunction of the inhibitory glycine receptor. *Chem. Biol.* **11**, 1339–1350 (2004).

6. Papke, D. & Grosman, C. The role of intracellular linkers in gating and desensitization of human pentameric ligand-gated ion channels. *J. Neurosci. Off. J. Soc. Neurosci.* **34**, 7238–7252 (2014).

7. Gielen, M., Thomas, P. & Smart, T. G. The desensitization gate of inhibitory Cys-loop receptors. *Nat. Commun.* **6**, 6829 (2015).

8. Amin, J., Gochman, A., He, M., Certain, N. & Wollmuth, L. P. *NMDA receptors require multiple pre-opening gating steps for efficient synaptic activity.* http://biorxiv.org/lookup/doi/10.1101/2020.06.09.142687 (2020) doi:10.1101/2020.06.09.142687.

9. Olsson, U. Confidence Intervals for the Mean of a Log-Normal Distribution. *J. Stat. Educ.* **13**, 3 (2005).
