# Supplementary material for: The desensitization pathway of GABAA receptors, one subunit at a time
Source: Nat Commun. 2020 Oct 23;11:5369. doi: 10.1038/s41467-020-19218-6 (PMC7585415; doi:10.1038/s41467-020-19218-6)
Supplement: Supplementary file 3 — Reporting Summary [file 41467_2020_19218_MOESM3_ESM.pdf]

nature research

Corresponding author(s): Marc Gielen

Last updated by author(s): Sep 16, 2020

Reporting Summary

Nature Research wishes to improve the reproducibility of the work that we publish. This form provides structure for consistency and transparency in reporting. For further information on Nature Research policies, see our Editorial Policies and the Editorial Policy Checklist.

Statistics

For all statistical analyses, confirm that the following items are present in the figure legend, table legend, main text, or Methods section.

n/a	Confirmed	
☐	☒	The exact sample size (*n*) for each experimental group/condition, given as a discrete number and unit of measurement
☐	☒	A statement on whether measurements were taken from distinct samples or whether the same sample was measured repeatedly
☒	☐	The statistical test(s) used AND whether they are one- or two-sided<br>*Only common tests should be described solely by name; describe more complex techniques in the Methods section.*
☒	☐	A description of all covariates tested
☒	☐	A description of any assumptions or corrections, such as tests of normality and adjustment for multiple comparisons
☐	☒	A full description of the statistical parameters including central tendency (e.g. means) or other basic estimates (e.g. regression coefficient) AND variation (e.g. standard deviation) or associated estimates of uncertainty (e.g. confidence intervals)
☒	☐	For null hypothesis testing, the test statistic (e.g. *F*, *t*, *r*) with confidence intervals, effect sizes, degrees of freedom and *P* value noted<br>*Give P values as exact values whenever suitable.*
☒	☐	For Bayesian analysis, information on the choice of priors and Markov chain Monte Carlo settings
☒	☐	For hierarchical and complex designs, identification of the appropriate level for tests and full reporting of outcomes
☒	☐	Estimates of effect sizes (e.g. Cohen's *d*, Pearson's *r*), indicating how they were calculated

Our web collection on statistics for biologists contains articles on many of the points above.

Software and code

Policy information about availability of computer code

Data collection	Software for electrophysiological data acquisition: Clampex (Axon pClamp 10.6.0.13) from Molecular devices.
Data analysis	Software for electrophysiological data analysis: Clampfit (Axon pClamp 10.6.0.13) from Molecular devices<br>Software for Markov-chain kinetic simulations: QUB Express 1.12.6 and QUB online. The online version is available at: https://qub.mandelics.com/online/#

For manuscripts utilizing custom algorithms or software that are central to the research but not yet described in published literature, software must be made available to editors and reviewers. We strongly encourage code deposition in a community repository (e.g. GitHub). See the Nature Research guidelines for submitting code & software for further information.

Data

Policy information about availability of data

All manuscripts must include a data availability statement. This statement should provide the following information, where applicable:

- Accession codes, unique identifiers, or web links for publicly available datasets
- A list of figures that have associated raw data
- A description of any restrictions on data availability

The data that support the findings of this study are available from the corresponding author upon reasonable request.

Field-specific reporting

Please select the one below that is the best fit for your research. If you are not sure, read the appropriate sections before making your selection.

☒ Life sciences ☐ Behavioural & social sciences ☐ Ecological, evolutionary & environmental sciences

For a reference copy of the document with all sections, see nature.com/documents/nr-reporting-summary-flat.pdf

Life sciences study design

All studies must disclose on these points even when the disclosure is negative.

Sample size

All electrophysiological experiments were peformed with Xenopus laevis oocytes from at least two different batches (i.e. oocytes obtained from ovaries of at least two different animals). Oocytes from different batches were recorded in fully independent set of experiments, separated by a time interval longer than a week. For each batch, oocytes expressing wild-type concatemers (CWT) were recorded as an internal quality control for the mutant receptors tested. This is the reason why the sample size for oocytes expressing wild-type concatemers (n = 40) is higher than the sample size for mutant concatemers (mean number of 7 +/- 2).
The minimal number of cells, i.e. 4 cells, was designed to assess the reproducibility of experiments: for each construct, we recorded at least 2 different cells on a given experimental day, and produced at least 2 different sets of independent data (i.e. using oocytes from at least 2 distinct animals). Low values for standard deviations and the low cell-to-cell variability provided arguments that the sample sizes were sufficient.

Data exclusions

Criteria for data exclusion were the following: 1) peak current size of insufficient magnitude for robust analysis; 2) prolonged current rise times (due to perfusion artifacts, oocyte placement in the recording chamber) precluding a reliable measurement of the fast desensitization component; 3) unstable current baseline, precluding a reliable measurement of the slow desensitization component.
Approximately 25% of electrophysiological recordings were excluded on these bases, across the entire range of constructs from CWT to C12345. Qualitative exclusion criteria were pre-established. However, they lead to relatively more data exclusion for strongly desensitizing mutants: 1) faster desensitization kinetics meant that prolonged current on-rates had more influence on the peak current amplitude; 2) smaller steady-state responses meant that unstability in the current baseline had more influence on the estimation of residual currents.

Replication

As an internal quality control, for each batch of Xenopus laevis oocytes used to express mutant concatemers, we recorded some oocytes expressing the wild-ype concatemers, thereby ensuring we could replicate recordings consistent with overall data for wild-type concatemers. For each construct, we performed at least 2 series of individual experiments (oocytes obtained from ovaries of two different animals), and recorded at least 2 cells for each series of recordings, yielding a total of at least 4 cells. All attempts were successful, i.e. each series of Xenopus oocytes DNA injection yielded experimental data used in the present work.

Randomization

Randomization is not relevant., as all oocytes were subjected to the same experimental treatment (application of 10mM GABA)

Blinding

Not relevant in our study: no conclusion is drawn from potential differences between constructs displaying similar phenotypes - please note that we didn't even perform any statistical analysis. Our kinetic analysis aims at explaining very large (up to 300 fold differences in weighted desensitization kinetics) and reproducible phenotypic differences, preventing from an effective blinding.

Reporting for specific materials, systems and methods

We require information from authors about some types of materials, experimental systems and methods used in many studies. Here, indicate whether each material, system or method listed is relevant to your study. If you are not sure if a list item applies to your research, read the appropriate section before selecting a response.

Materials & experimental systems

n/a	Involved in the study
☒	☐ Antibodies
☒	☐ Eukaryotic cell lines
☒	☐ Palaeontology and archaeology
☐	☒ Animals and other organisms
☒	☐ Human research participants
☒	☐ Clinical data
☒	☐ Dual use research of concern

Methods

n/a	Involved in the study
☒	☐ ChIP-seq
☒	☐ Flow cytometry
☒	☐ MRI-based neuroimaging

Animals and other organisms

Policy information about studies involving animals; ARRIVE guidelines recommended for reporting animal research

Laboratory animals

We used oocytes from Xenopus laevis. Ovaries were obtained from CRB Xenopes (UMS 3387, CNRS / Université de Rennes, Rennes, France). No animals were handled by the authors.

Wild animals	No wild animals were used in the study.
Field-collected samples	No field-collected samples were used in the current study.
Ethics oversight	For the supply of Xenopus laevis ovaries, authorization was granted to CRB Xenopes by the Direction Départementale de la Cohésion et de la Protection des Populations, Santé et Protection Animales et Végétales, at the Préfecture d'Ille-et-Vilaine, under the number A 35-238-42.

Note that full information on the approval of the study protocol must also be provided in the manuscript.
